# Supplementary material for: “Guilt by association” is not competitive with genetic association for identifying autism risk genes
Source: Sci Rep. 2021 Aug 5;11:15950. doi: 10.1038/s41598-021-95321-y (PMC8342445; doi:10.1038/s41598-021-95321-y)
Supplement: Supplementary file 1 — Supplementary Information 1. [file 41598_2021_95321_MOESM1_ESM.pdf]

Supplementary File: Can machine learning aid in identifying disease genes? The case of autism spectrum disorder

Margot Gunning[1,2,3], and Paul Pavlidis[1,2,4*]

[1] Michael Smith Laboratories, University of British Columbia, Vancouver BC, V6T 1Z4, Canada

[2] Department of Psychiatry, University of British Columbia, Vancouver BC, V6T 1Z4, Canada

[3] Graduate Program in Bioinformatics, University of British Columbia, Vancouver BC, V6T 1Z4, Canada

[4] Djavad Mowafaghian Centre for Brain Health, University of British Columbia, Vancouver BC, V6T 1Z4, Canada

* To whom correspondence should be addressed. Tel: +1 (604) 827-4157; Email: paul@msl.ubc.ca

Supplementary Tables

Supplementary Table 1: SFARI-HC genes in other TADA studies not found to be significantly associated with ASD in Satterstrom

Study	nGenes	Genes
DeRubeis	5	CUL3, KATNAL2, NAA15, RELN, TRIO
Sanders	7	CUL3, KATNAL2, NAA15, NCKAP1, NLGN3, TRIO, WDFY3
iHart	5	CUL3, DDX3X, KATNAL2, NCKAP1, WDFY3

Supplementary Table 2: Shared SFARI-HC genes across TADA studies.

Study	nGenes	Genes
TADA	17	ADNP, ANK2, ARID1B, ASH1L, BCL11A, CHD8, DYRK1A, GRIN2B, KMT2C, KMT5B, MYT1L, POGZ, PTEN, SCN2A, SETD5, SYNGAP1, TBR1

Supplementary Table 3: Seven novel-HC genes now considered to be SFARI-HC genes.

Study	nGenes	Genes
Spark	2	BRSK2, NR4A2
iHart	1	DDX3X
Satterstrom	4	HECTD4, RFX3, RORB, TAOK1

Supplementary Table 4: Summary statistics for forecASD versions on novel-HC genes. AUROC: Area under the receiver operator characteristic curve; P20R: precision at 20% recall; P43R: precision at 43% recall. Values in parentheses are the upper and lower 95% confidence interval bounds.

**forecASD version**	**AUROC**	**P20R (%)**	**P43R (%)**
Redo	0.89 (0.84, 0.94)	7.13 (4.63, 11.21)	5.56 (3.24, 9.38)
NoClass	0.88 (0.84, 0.93)	8.51 (3.34, 17.1)	4.39 (2.34, 6.14)
NoClassPPI	0.87 (0.82, 0.92)	6.82 (4.49, 9.87)	4.55 (2.46, 7.60)
NoClassPPIBS	0.85 (0.80, 0.90)	6.14 (2.88, 9.73)	2.96 (1.55, 4.92)
PPIOnly	0.69 (0.63, 0.74)	0.85 (0.40, 1.76)	0.52 (0.38, 0.81)
BrainSpanOnly	0.69 (0.63, 0.76)	1.04 (0.81, 2.51)	0.92 (0.54, 1.53)

Supplementary Table 5: Summary statistics for forecASD versions on SFARI-HC genes, similar to supplementary table 4. A '*' for P43R indicates a tie at recall of 20/43% of gene set

**forecASD version**	**AUROC**	**P20R (%)**	**P43R (%)**
Redo	0.94 (0.92, 0.95)	53.56 (38.23, 77.32)	31.80 (23.85, 40.22)
NoClass	0.92 (0.90, 0.94)	59.18 (36.19, 77.27)	26.09 (15.69, 38.38)
NoClassPPI	0.89 (0.87, 0.92)	63.04 (30.00, 79.17)	21.19 (12.17, 31.29)
NoClassPPIBS	0.87 (0.85, 0.89)	56.34 (34.78, 77.11)	10.56 (7.05, 19.74)
PPIOnly	0.79 (0.76, 0.83)	8.76 (5.44, 13.95)	5.12 (4.16, 6.55)*
BrainSpanOnly	0.80 (0.77, 0.83)	5.64 (4.41, 8.14)	3.86 (2.87, 5.75)*

Supplementary Text

Here we provide more detailed descriptions of the gene prioritization methods we examined in this study, and summarize the performance evaluations and claims from the source publications.

Genetic association approaches

TADA: An increasingly common test for association between rare genetic variation and disease, particularly in the field of autism genetics research, is the Transmission and *De Novo* Association Analysis (TADA) test (De Rubeis et al., 2014; Feliciano et al., 2019; He et al., 2013; Ruzzo et al., 2019; Sanders et al., 2015; Satterstrom et al., 2020). TADA employs a gene-level approach by allowing for recurrence of multiple types of variants to be collapsed in order to maximize power to find risk genes (He et al., 2013). The test uses data from *de novo* and/or inherited variants identified by large scale sequencing studies of simplex and multiplex families and case-control cohorts. Using this data, TADA builds a likelihood model based on allele frequency, relative risks of different classes of variation, and mutation rates to estimate a gene's likelihood of being involved in the phenotype. TADA can be seen as a "family of methods" (fundamentally, statistical models) because it can be applied using only *de novo* variation (TADA-Denovo) or using *de novo*, inherited and case-control variation (TADA), and requires parametrization of multiple variants (He et al., 2013). One implication of the lack of well-established and validated methods for gene-level association studies of rare variants it that two studies of the same cohort can get different results, even if they both use a method labeled "TADA." Most recent ASD sequencing studies employing a TADA test provide an association score for each gene in the genome, and identify a subset a genes significantly associated with ASD under their model, at some expected false discovery rate (De Rubeis et al., 2014; Ruzzo et al., 2019; Sanders et al., 2015; Satterstrom et al., 2020). The genome-wide association scores allow us to compare prioritization of ASD risk gene candidates based on genetic association to other genome-wide prioritization scores based on other types of non-genetics data.

Iossifov LGD: First, they calculated a gene's "vulnerability score" based on: 1) A likelihood model of expected LGD variants in a typical gene built from synonymous variation data in the parents of the SSC and the control neurotypicals; and 2) The proportion of causal ASD genes estimated from the ascertainment differential for LGD variation between affected probands and unaffected sibling controls (Iossifov et al., 2015). By combining the "vulnerability score" with the observed load of LGD variants in proband WES data, they created a heuristic prioritization score for ASD genes (Iossifov et al., 2015).

GBA ML methods

Studies in this class do not use information from ASD genetic association studies, but they use machine learning algorithms to distinguish ASD from non-ASD risk genes using other types of non-genetics data.

Princeton (Krishnan et al., 2016) used a support vector machine (SVM) trained on a human brain-specific functional interaction network built from multiple protein-protein interaction databases, gene expression datasets, and other regulatory and genetic and chemical perturbation data (Greene et al., 2015). Their training labels included 549 positive genes weighted by the

strength of evidence of association with ASD (E1,2,3,4), and a set of 1189 manually curated non-mental health disease genes. Their feature space was a gene-gene matrix where the cells represented the probability of a gene-gene interaction calculated from their brain-specific functional interaction network. Using their gene-gene matrix, and their ASD-positive and ASD-negative training gene sets, they fit a linear SVM with a penalty parameter to control misclassification of their evidence-weighted labels (i.e. lower misclassifications of E1, high-confidence labels). They ran 5-fold cross-validation 50 times on different subsets of their evidence-weighted training labels and found that the model with all evidence-weighted labels had the best performance for separating positive and negative genes. In theory, the SVM fit a linear plane in the high-dimensional feature space which was able to maximize the separation between positive and negative training genes. For each candidate gene, the distance between the candidate gene and discriminant hyperplane (i.e. prediction from computing the linear kernel function) was converted to a probability using regression; an average probability was taken across each of the 5 cross-validation folds. Prediction scores were provided for 25 825 genes, and they identified their top decile of genes as likely ASD risk gene candidates. Their published evaluation and validation of their ranking system included: 1) Calculating enrichment of genes with *de novo* mutations in independent ASD sequencing studies in their top decile (Sanders et al. (2012), O'Roak et al. (2012), Iossifov et al. (2012), Neale et al. (2012), Iossifov et al. (2014), and De Rubeis et al. (2014)); 2) Calculating enrichment of experimentally determined targets of ASD-related proteins and pathways, such as FMRP and MAPK signalling, in their top decile; and 3) Calculating enrichment of genes found to be associated with intellectual disability, schizophrenia, and other developmental disorders in their top decile. From their main evaluation, they found that their evidence-weighted labels had significantly better performance during cross validation than other combinations of training labels, and that there was significant enrichment of genes found to have *de novo* likely damaging variation in independent ASD sequencing studies in their top decile of genes. Overall, they concluded that their method was able to prioritize many new ASD candidate risk genes, and claimed that their top ranked genes had the potential to speed up ASD gene discovery.

FRN (Duda et al., 2018) used a random forest classifier with a brain-specific functional interaction network. They built their network from human, rat and mouse gene expression datasets from non-cancer related brain experiments, multiple protein-protein interaction databases, and protein docking and phenotype annotations. They utilized 143 ASD genes from SFARI 1, 1S, 2, 2S and Sanders as positive training labels, and 1176/1189 of the negative non-mental health genes from Princeton. Their feature space was a gene-gene matrix where the cells represented the probability of a gene-gene interaction based on their brain-specific functional interaction network. Using their gene-gene matrix, and their ASD-positive and ASD-negative training gene sets, they trained 5 different machine learning models with 5-fold cross-validation, and found that their random forest model had the best performance based on the average AUROC from the 5-folds. Random forests are built from multiple decision trees which segment the feature matrix into a number of simple regions by recursive binary splitting. In each decision tree of a random forest, each split in each tree uses a random sample of features, and at each successive split, the best splitting rule is chosen so that the two new regions are as pure as possible. In other words, the feature and its threshold which give the best separation between the positive and negative training data is chosen at each split point in each tree. The leaves at the bottom of a decision tree are called terminal nodes. Predictions are made for candidate items

(genes) based on which decision path it follows, and the proportion of positive and negative training observations in the terminal node. In other words, after allowing a candidate gene to follow a decision path and entre a terminal node, if the majority of the genes in the terminal node are positive training genes, the candidate gene will be predicated as a positive. Prediction scores were provided for 21,114 genes, and they identified their top 2,111 genes as likely ASD risk gene candidates. Their published evaluation and validation of their ranking system included: 1) Calculating the enrichment of genes with recurrent and non-recurrent *de novo* LoF mutations in ASD probands and unaffected siblings from the SSC (Iossifov et al. (2014)) and MSSNG (Yuen et al. (2017)) in their top decile; and 2) Calculating the enrichment of genes found to be involved in Alzheimer's disease, Parkinson's disease and ataxia. From their evaluation, they found significant enrichment of genes with *de novo* LoF mutations in SSC and MSSNG probands in their top decile, and an absence of significant enrichment for genes involved in other brain-related disorders. Overall, they concluded that their method predicted genes with evidence of ASD association and was able to propose numerous novel genes they claimed had a high likelihood of contributing to ASD.

DAMAGES (C. Zhang & Shen, 2017) used cell-type specific expression data from 24 mouse central nervous system cell types from 6 regions, and measures of constraint against LoF and missense variation from ExAC to try to identify ASD risk genes. First, they created a DAMAGES (D) score built from gene expression profiles of 145 genes found to have *de novo* LGD variants in probands and unaffected siblings from Iossifov et al. (2012), Neale et al. (2012), O'Roak et al., (2012) and Sanders et al. (2012) using Principal Component Analysis (PCA). Regression analysis was used to evaluate how each principal component from the PCA analysis was able to predict a gene's variation source as proband or sibling control. Next, they used logistic regression to combine the D score with measures of constraint against LoF and missense variation to create an ensemble score (E). Their training labels for their logistic regression classifier were 36 genes found to have 2 or more *de novo* LGD mutations in ASD probands, and 156 genes with only 1 or more *de novo* LGD mutations in sibling controls. Their feature space consisted of the D score (PCA-based gene expression profiles) and ExAC constraint scores. They used logistic regression to estimate the effect size of each feature, and then calculated an ensemble (E) score for each candidate gene predicting its likelihood of being a haploinsufficient ASD gene. The mouse genes they used to create the D score were mapped to human orthologs so the constraint scores could be added to create the E score. They identified the top 117 genes by E score as likely ASD risk gene candidates. We kept E scores for 15 881 genes with single, unambiguous mappings to human genes. Their published evaluation and validation of their ranking system included: 1) Calculating enrichment of genes with LGD mutations from sequencing studies published after 2012 (De Rubeis et al. (2014), Iossifov et al. (2014)), and 438 SFARI genes by category (S,2,3,4,5,6) in the top ranking of the D score; 2) Comparing the D score and Ensemble score to constraint measures alone, and ranks provided by Princeton by calculating a modified precision recall statistic. From their evaluations, they found enrichment of genes found to have *de novo* likely damaging variation in independent ASD sequencing studies, and that their method have favourable performance compared to other studies. Overall, they concluded that their gene expression signatures reflected haploinsufficiency in ASD, and claimed that it was able to predict whether or not likely damaging variants confer increased risk to ASD.

RF_Lin (Lin et al., 2018) employed a random forest classifier using gene-level constraint measures from ExAC and a weighted network built from BrainSpan and InWeb protein-protein interaction data as features (Li et al., 2017; Miller et al., 2014). Their training labels are the same employed in the FRN method above. See ASD_frn for description of random forests. In theory, their random forest was able to split the feature space (constraint, weighted network information) into regions which could separate their positive training genes from the negative training genes, and thereby predict which candidate genes were the most similar to positive training genes. Prediction scores were provided for 17 099 genes, and they identified their top 2 089 genes as likely ASD risk gene candidates. They did not provide scores for their training labels. Their published evaluation and validation of their ranking system included: 1) Calculating enrichment of genes found to have *de novo* LoF or missense mutations from 2517 SSC families (Iossifov et al. (2014)) and MSSNG (Yuen et al. (2017)) in their top decile; 2) Comparing their ranking system to ExAC pLI, Iossifov, Sanders, Princeton and DAMAGES by calculating decile enrichment of 130 SFARI category 3 genes, and 43 genes with recurrent *de novo* LoF mutations identified in Stessman et al. (2017), Wang et al., (2016), Yuen et al., (2017), and Li et al., (2017); and 3) Comparing their ranking system to ExAC pLI, Iossifov, Sanders, Princeton and DAMAGES by calculating the AUROC with their labelled and unlabelled data, with the 130 SFARI category 3 genes, and with the 43 genes with recurrent *de novo* LoF mutations. From their evaluations, they found significant enrichment of genes with *de novo* LoFs in SSC and MSSNG probands, and that their method showed higher enrichment of 173 candidate ASD genes in their top decile compared to other methods. Overall, they concluded that their method demonstrated that spatiotemporal gene expression and constraint metrics predicted ASD risk genes, and claimed that their method provided many new candidate genes with strong evidence of contributing to ASD.

PANDA (Y. Zhang et al., 2020) is a graph neural network type classifier. They built an unweighted and undirected human molecular interaction network from experimentally documented physical protein interactions using data from a previously established protein-protein interaction network (Menche et al., 2015), and BioGRID (Oughtred et al., 2019). Their training labels included 760 autism-associated genes from SFARI Gene 2.0 (Abrahams et al., 2013), and Online Mendelian Inheritance in Man (Hamosh et al., 2005) weighted by confidence of association (0.5, 0.75, 1.0), and 1102 non-autism associated genes from FRN and Princeton above. They training their algorithm using five-fold cross-validation. The human molecular interaction network was represented as a graph whereby the genes were nodes, and their interactions were edges. They described the local structural properties of each node using six node centrality measures and calculated how often a node appeared in 69 different orbits (distinct positions of nodes) in 4- and 5- node graphlets (connected, nonisomorphic induced subgraphs). Next, a local transition function aggregated all the network properties of each node, and its direct neighbours in ten dimensions to obtain node embeddings (10-dimenstional vector describing each node). Using these 10-dimensional spatial representations, a global output function was used to predict the class label of each node (1=autism-associated, 0=no autism-association). In addition, they used a sigmoid function to transform each prediction into a probability and computed a loss function to penalize embeddings that encode neighbours very differently. They had four layers to their classifier, meaning their local transition and global output functions were implemented as four feed-forward neural networks. Prediction scores were provided for 23 472 genes, and they identified an "autism subnetwork" of 2 346 genes. Their published evaluation

and validation of ranking system included: 1) Evaluation of the classification performance of PANDA by calculating sensitivity, specificity, classification accuracy, precision@k (proportion of positive autism-genes in top-k ranked list), and comparing to three other types of machine learning algorithms; 2) Calculating enrichment of genes with *de novo* likely disrupting mutations from probands and their unaffected siblings an independent sequencing study (Iossifov et al., 2014) in their top decile of genes; 3) Calculating the specificity of PANDAs rankings to ASD by looking at the distribution of Alzheimer's, Parkinson's and Epilepsy genes in their rankings; 4) Investigating their unlabelled top-ranked genes for potential association with ASD; and 5) Identifying an "autism subnetwork" from the human molecular interaction network made up of 2 346 genes. From their evaluations, they found that PANDA was the best performing type of machine learning algorithm, and that their top decile of genes from ranked gene list showed significant enrichment for recurrent and non-recurrent proband DN-LGDs. Further, they found that their rankings specific to ASD due to lack of significant enrichment of other disease genes in their top decile. Overall, they concluded that PANDA was able to search the graph space to find genes with similar topological properties or indirect connections to known autism genes, and predict which are the most likely to be autism genes themselves.

Hybrid Genetics-GBA machine learning studies

The studies in this section used a combination of ASD-specific features and other features to build their models. The ASD-specific features come from genetic association data in the studies described above. Information from the two classes of features are integrated prior to training a machine learning algorithm to distinguish ASD from non-ASD risk genes, using high-confidence ASD genes from genetic association studies as their positive training set.

DAWN (Liu et al., 2014) targeted their search to genes found to be expressed in the prefrontal and motor-somatosensory (PFC-MSC) neocortex during the 10-24 weeks post-conception phase based on previous findings from Willsey et al.(2013). Willsey et al., (2013) found that the PFC-MSC from 10-24 weeks post-conception was a potential nexus for risk based on coalescence of gene expression during that time. They built a co-expression network from BrainSpan data of the selected regions and time points using Weighted Gene Co-expression Network Analysis (WGCNA) and overlaid genetic association statistics from a TADA model to identify ASD risk gene candidates (Langfelder & Horvath, 2008; Liu et al., 2014). The TADA scores utilized were calculated from rare *de novo* likely damaging variants, rare transmitted likely damaging variants and case-control likely damaging variants from multiple sources, including Iossifov et al. (2012), Neale et al. (2012), O'Roak et al. (2012), Sanders et al. (2012), and the ARRA Autism Sequencing Consortium, among others. They used unsupervised model-based clustering and a hidden Markov random field to model the correlation of genetic association scores across the co-expression network to identify co-expressed nodes with high genetic evidence of association with ASD ("network ASD genes"). Next, they used a false discovery rate procedure to determine which of the "network ASD genes" were most likely to contribute to ASD ("risk ASD genes", rASD genes). They provided prediction scores for the 10 233 genes in the network which had exome data. They identified their top 127 genes (FDR < 0.05) to be likely ASD risk gene candidates. Their published evaluation and validation of their ranking system included: 1) Two permutation dilution experiments were conducted whereby the signal from genetic association or co-expression data was diluted to determine the sensitivity of the rASD gene list to either signal;

and 2) Calculation of enrichment of *de novo* LoF mutations identified in a targeted sequencing study of 44 ASD candidate genes in 2448 ASD trios in their 127 rASD genes. From their evaluation experiments, they found that DAWN was sensitive to both the TADA signal and the co-expression signal, and that DAWN was able to identify genes found to have more *de novo* LoF variants in ASD probands. Overall, they concluded that there was a high likelihood that DAWN had identified true ASD risk genes.

forecASD (Brueggeman et al., 2020) is a stacked random forest ensemble classifier. They built the first layer from BrainSpan spatiotemporal gene expression data (Miller et al., 2014) and a protein-protein interaction matrix built from STRING (Szklarczyk et al., 2019). The second layer was built from the scores from layer 1, and scores from other studies, all of which are included in my study (Princeton, DAWN, DAMAGES, De Rubeis and Sanders). Their training data included 76 SFARI high-confidence ASD genes, and 1000 random non-SFARI genes. See ASD_frn for description of random forests. In theory, each random forest layer they built was able to split the feature space (BrainSpan, STRING, genome-wide ASD prediction sores) into regions which could separate their positive from their negative training genes, allowing for candidate gene predictions to be made based on shared/similar associations to the positive training genes in the feature space. Prediction scores were provided for 17 957 genes, and they identified their top 1787 as likely ASD risk gene candidates. Their published evaluation and validation of their ranking system included: 1) Calculating enrichment of genes with *de novo* likely disrupting mutations in MSSNG (Yuen et al., (2017)) and Spark (unpublished at time of forecASD development) probands, SFARI 3, 4, 5 and syndromic genes, and gene targets of CHD8 and FMRP in their top decile of ranks; 2) Calculating the AUROC for their score, and scores from Princeton, DAMAGES and Sanders on SFARI category 1 and 2 genes, and SFARI category 3 genes; 3) Calculating enrichment of genes with *de novo* likely disrupting mutations in MSSNG (Yuen et al. (2017)) and Spark (unpublished at time of forecASD development) probands in the top decile of the scores from Princeton, DAMAGES and Sanders for comparison; and 4) Fitting logistic regression models to assess how much forecASD is adding to genetic TADA signals. From their evaluations, they found their method was better able to classify SFARI 1 and 2 genes and SFARI 3 genes, and they showed greater enrichment in their top decile for genes found to have recurrent *de novo* likely damaging variants in Spark and MSSNG probands compared to other studies. Further, they concluded that forecASD was able to provide biological context to TADA genetic signals important for prioritization. Overall, they concluded that their method was able to generalize to new data and claimed that they had created a valuable framework for combing both genetics and non-genetics data to prioritize ASD risk gene candidates which will be useful for when ASD gene discovery by genetic association slows.
